# Population structure and hybridization under contemporary and future climates in a heteroploid foundational shrub species (*Artemisia tridentata*)

**DOI:** 10.3389/fpls.2023.1155868

**Published:** 2023-05-22

**Authors:** Lukas P. Grossfurthner, Elizabeth R. Milano, Paul A. Hohenlohe, Lisette P. Waits, Bryce A. Richardson

**Affiliations:** ^1^ Bioinformatics and Computational Biology Graduate Program, University of Idaho, Moscow, ID, United States; ^2^ Department of Biological Sciences, University of Idaho, Moscow, ID, United States; ^3^ Rocky Mountain Research Station, United States Department of Agriculture (USDA) Forest Service, Moscow, ID, United States; ^4^ Department of Fish and Wildlife Sciences, University of Idaho, Moscow, ID, United States

**Keywords:** foundational shrub, ecological divergence, heteroploid dataset, sagebrush, ddRAD-sequencing, ecotones

## Abstract

Current and past climatic changes can shift plant climatic niches, which may cause spatial overlap or separation between related taxa. The former often leads to hybridization and introgression, which may generate novel variation and influence the adaptive capacity of plants. An additional mechanism facilitating adaptations to novel environments and an important evolutionary driver in plants is polyploidy as the result of whole genome duplication. *Artemisia tridentata* (big sagebrush) is a landscape-dominating foundational shrub in the western United States which occupies distinct ecological niches, exhibiting diploid and tetraploid cytotypes. Tetraploids have a large impact on the species’ landscape dominance as they occupy a preponderance of the arid spectrum of *A. tridentata* range. Three distinct subspecies are recognized, which co-occur in ecotones – the transition zone between two or more distinct ecological niches – allowing for hybridization and introgression. Here we assess the genomic distinctiveness and extent of hybridization among subspecies at different ploidies under both contemporary and predicted future climates. We sampled five transects throughout the western United States where a subspecies overlap was predicted using subspecies-specific climate niche models. Along each transect, we sampled multiple plots representing the parental and the potential hybrid habitats. We performed reduced representation sequencing and processed the data using a ploidy-informed genotyping approach. Population genomic analyses revealed distinct diploid subspecies and at least two distinct tetraploid gene pools, indicating independent origins of the tetraploid populations. We detected low levels of hybridization (2.5%) between the diploid subspecies, while we found evidence for increased admixture between ploidy levels (18%), indicating hybridization has an important role in the formation of tetraploids. Our analyses highlight the importance of subspecies co-occurrence within these ecotones to maintain gene exchange and potential formation of tetraploid populations. Genomic confirmations of subspecies in the ecotones support the subspecies overlap predicted by the contemporary climate niche models. However, future mid-century projections of subspecies niches predict a substantial loss in range and subspecies overlap. Thus, reductions in hybridization potential could affect new recruitment of genetically variable tetraploids that are vital to this species’ ecological role. Our results underscore the importance of ecotone conservation and restoration.

## 1 Introduction

Climate is among the main factors shaping the distribution of plant assemblages across landscapes, and climatic changes have major implications for the evolution and distribution of organisms. In the Pleistocene, climatic oscillations triggered range shifts, reticulate evolution, and whole genome duplication (WGD) events in plant species (Comes and Kadereit, 1998; Kreiner et al., 2017). During glacial periods, plant climatic niches shifted towards lower latitudes, lower altitudes, or to disjunct refugial areas within mountain ranges which – in some regions – were additionally fragmented by extensive pluvial lakes (Davis, 1999; Holderegger and Thiel-Egenter, 2009; Houston et al., 2014). Populations in refugia were often small, leading to divergent processes that caused pockets of endemism which had the potential to trigger the origin of new taxa (Roberts and Hamann, 2015). Subsequent post-glacial range expansions caused secondary contact, hybridization between divergent lineages, and stimulated reticulate evolution – either at the homoploid level (= same ploidy) or, after WGD, leading to allopolyploid formation. Conversely, WGD events within populations were triggered by the increased production of unreduced gametes which had been stimulated by temperature oscillations (autopolyploidy; Ramsey and Schemske, 1998; Kreiner et al., 2017). Here we apply the term ‘homoploid’ to any instance of interbreeding between two taxa of the same ploidy, regardless of the evolutionary relationships or outcome.

Contemporary increases in mean annual temperatures and changing precipitation patterns may cause habitat loss (Breshears et al., 2005) and typically shift plant climatic niches towards higher latitudes and altitudes (Parmesan and Yohe, 2003; Kelly and Goulden, 2008). Consequently, climatically induced range shifts may cause spatial overlap and separation between related taxa (i.e., sympatry and allopatry). Such processes could lead to differential opportunities for hybridization and could have major implications for environmental adaptation and speciation (Chunco, 2014; Ryan et al., 2018).

The *Artemisia tridentata* Nutt. (big sagebrush) subspecies complex provides an excellent study system to address the evolutionary consequences of climatic change on divergence into ecologically differentiated taxa, hybridization, and WGD. *Artemisia tridentata* is a perennial, and wind-pollinated shrub which occupies distinct ecological niches throughout the intermountain region in the western United States. This species consists of three widespread subspecies: *Artemisia tridentata* subsp. *tridentata* Beetle (hereinafter subsp. *tridentata*, basin big sagebrush) occurs on deep, well-drained soils; *A. tridentata* subsp. *wyomingensis* Beetle and Young (hereinafter subsp. *wyomingensis*, Wyoming big sagebrush) grows in similar climates as subsp. *tridentata* but is adapted to shallow soils; *A. tridentata* subsp. *vaseyana* (Rydb.) Beetle (hereinafter subsp. *vaseyana*, mountain big sagebrush) occupies cooler and moderately moist habitats at higher elevations (Mahalovich and McArthur, 2004; Shultz, 2009; Brabec et al., 2017). Both subsp. *tridentata* and subsp. *vaseyana* are predominantly diploid (2*n*=2*x*=18), but tetraploid individuals (2*n*=4*x*=36) occur infrequently (McArthur and Sanderson, 1999; Chaney et al., 2017). Despite being strictly tetraploid (2*n*=4*x*=36), subspecies *wyomingensis* is very common in basins. All three subspecies are ecologically, cytologically, and chemically distinct (McArthur and Sanderson, 1999; Jaeger et al., 2016; Brabec et al., 2017). Nuclear amplicon genetic analysis also supports the validity of described subspecies – particularly at the diploid level (Richardson et al., 2012). Despite strong evidence for differentiation, all of the aforementioned studies also reported intermediate forms, indicating hybridization where subspecies co-occur (Goodrich et al., 1999; McArthur and Sanderson, 1999; Shultz, 2009; Richardson et al., 2012; Jaeger et al., 2016). Co-occurrence is particularly high in basin-mountain ecotones, supporting extensive hybrid formation (Sanderson and McArthur, 1999) as identified by intermediate phenotypic traits (McArthur et al., 1988). Genomic confirmation and knowledge of the extent of hybridization between subspecies at different ploidy levels on a larger spatial scale are lacking, however, and it is unclear how climate change may affect opportunities for further hybridization.

Here, we investigate the relationships among *A. tridentata* subspecies, aiming to assess the roles of hybridization and WGD on lineage divergence. To this end, we constructed ecological niche models – based on previously published literature (Still and Richardson, 2015) and herbarium specimens – to identify regions of co-occurring subspecies. We then sampled five transects along the regions with overlapping ecological niches, following environmental (i.e., elevational) gradients, in the intermountain region of the western United States. We collected *A. tridentata* specimens at multiple plots along each transect, aiming to represent distinct parental subspecies and potential hybrids. To identify genetic variants, we applied cytological methods (flow-cytometric ploidy determination), and double-digest restriction-site associated DNA sequencing (ddRADseq; Parchman et al., 2012), coupled with a ploidy-informed genotype likelihood approach (Blischak et al., 2018), which has been proven successful in analyzing polyploid species complexes (Brandrud et al., 2020; Ahmad et al., 2021). Specifically, we (i) evaluate range shifts under predicted future climate conditions and test whether the overlapping range between subspecies is predicted to contract or expand under future climate conditions. We further test (ii) whether the three subspecies of *A. tridentata* are genetically distinct lineages as implied by taxonomy and (iii) whether there is hybridization between subspecies at the homoploid, and (iv) heteroploid (= different ploidy) level, as suggested by intermediate morphological and chemical traits. Lastly, we (iv) identify potential parents of the tetraploid lineages and whether they are of auto- or allopolyploid origin.

We predict two distinct diploid subspecies and a heterogeneous tetraploid lineage. We expect hybridization to occur in ecotones – largely between diploid subspecies – with a smaller fraction of introgressed tetraploids. We hypothesize multiple origins of the tetraploids as a result of hybridization followed by WGD (=allopolyploidy). Lastly, we predict a range contraction and spatial decoupling of subspecies, causing a reduction of hybridization potential under future climate scenarios.

## 2 Materials and methods

### 2.1 Climate niche modeling under contemporary and future climate conditions

To model *A. tridentata* subspecies climate suitability and overlapping niches (i.e., hybrid zones), we used a presence-background random forest classification approach for the current and two future climate scenarios. We generated two climate niche models: one for subsp. *vaseyana*, which generally occupies a more montane ecological niche, and a second for subsp. *wyomingensis* and subsp. *tridentata* which occupy lower basins. Subspecies *wyomingensis* and *tridentata* were combined because of their largely sympatric distributions at the spatial scale resolution of our modeling (ca. 1-km^2^; Mahalovich and McArthur, 2004; Still and Richardson, 2015).

*Artemisia tridentata* presence data were gathered from Bureau of Land Management (BLM) Assessment, Inventory, and Monitoring (AIM) Strategy data (https://www.blm.gov/aim/strategy, accessed: October 2022), confirmed herbaria records, and known survey points (Still and Richardson, 2015). Background points were generated by sampling six random points on a 0.2-degree resolution raster grid using the randomPoints function in the dismo 1.3-5 package (Hijmans et al., 2021) in R 4.2.1 (R Core Team, 2022). Four-km^2^ annual and seasonal climate surfaces, downscaled to 1-km^2^, were obtained from ClimateNA (Wang et al., 2016) for the 30-year-normal current conditions (1980–2010) and two future climate scenarios from ensembled global climate models; mid-century (2050) representative concentration pathway (RCP)4.5 and 2050 RCP8.5. The RCP4.5 describes a moderate scenario, with temperature increases ranging from 0.9°C to 2°C, whereas the RCP8.5 is the highest emission scenario with predicted temperature increases ranging from 1.4°C to 2.6°C by mid-century, respectively (IPCC, 2014). Point estimates for each presence/background location were extracted from the climate layers using the extract function in the raster 3.5-21 R package (Hijmans, 2022). Climate variables for each model were filtered to remove co-linear relationships and correlations |r| > 0.7.

We utilized the randomForest function of the randomForest 4.7-1.1 R package (Liaw and Wiener, 2002) to train (90% of data) and validate (10% of data) each of the two datasets, respectively. Parameters for each model were optimized to minimize the out-of-bag (OOB) error by permeating the number of trees (ntree) and the number of randomly sampled variables (mtry) parameters for the training dataset. The final parameters were mtry=3 and ntree=1500, for each model, respectively. Subsequently, each model was assessed for accuracy and specificity using the validation datasets. Species distributions for the current and future climate scenarios were predicted on the currently known extent of *A. tridentata* using a custom wrapper for the predict function of the randomForest R package. Climate layers were cropped from -130°W to -100°W longitude and 26°N to 60°N latitude, predictions were output in ASCII format, and normalized vote counts > 0.6 were classified as a positive presence prediction. Overlapping regions were identified by merging the two climate niche models for each climate scenario, respectively.

### 2.2 Sampling and DNA isolation

**Figure 1:**
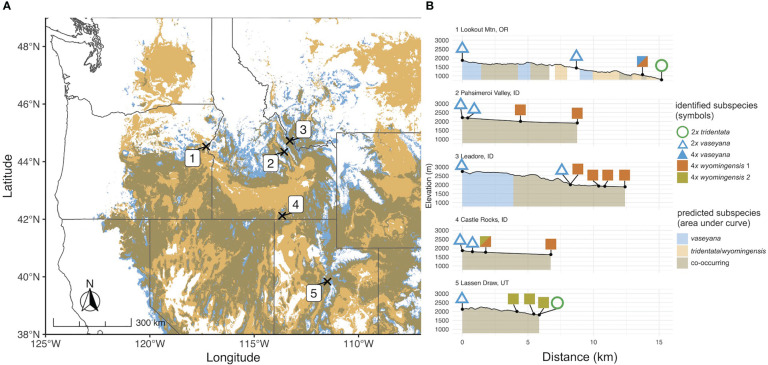
**(A)** Transect locations in the study region on the predicted contemporary distribution of *A*. *tridentata* subspecies based on the climate niche model. **(B)** Elevation profiles of sampled transects. Numbers correspond to sampling locations; symbols correspond to ploidy and the genetically identified subspecies; coloration under the elevation profile corresponds to the predicted subspecies climatic niche.

Plant material was collected at five elevational transects across the western United States based on the overlapping subspecies distribution as identified by the climate niche model (**Figure 1A**). At each transect we sampled four or five plots representing two parental habitats and at least two putative hybrid habitats, with 20 individuals per plot, respectively. The parental plants were identified using available determination keys (Shultz, 2009; Hitchcock et al., 2018) as well as morphological and chemical traits (Richardson et al., 2018). Altogether, 420 samples were collected: 40 of which were identified as subsp. *tridentata*, 60 as subsp. *wyomingensis* and 100 as subsp. *vaseyana*. The remaining 220 samples were considered to be potential hybrids due to intermediate ecological, morphological or chemical traits. Vouchers for each sample are deposited in the Stillinger Herbarium of the University of Idaho (ID). A comprehensive list of collected samples including their corresponding metadata is provided in **Table S1**. All plant material was immediately dried and stored in silica gel. Additionally, fresh plant material was collected for each sample and stored in a dry plastic bag at 4°C for subsequent ploidy analysis.

For DNA extraction, 20 µg of silica-dried leaf tissue was ground for 1 min with a ball mill (Retsch MM400, Retsch, Haan, Germany). Secondary metabolites were removed following an overnight incubation at 4°C in 700 µl sorbitol wash buffer (Inglis et al., 2018). Two additional sorbitol washing steps were performed after a 10-minute incubation on ice and centrifugation at 10,000 rpm. DNA extraction was performed using CTAB, following Inglis et al. (2018). DNA was quantified using assay kits (Qubit dsDNA HS and BR Assay Kits, Thermofisher Scientific, Waltham, MA, USA) following the manufacturer’s protocol.

### 2.3 Ploidy analysis

About 1-cm^2^ of fresh leaf tissue was chopped in 330 µl Nuclei Extraction Buffer (Sysmex, Kobe, Japan) together with dried leaf tissue from *Acer negundo* (0.535pg/1C, Loureiro et al., 2007; 0.765pg/1C, Contreras and Shearer, 2018), the taxon which was used as internal standard. Cell wall remnants were removed by filtering the isolate through a 30-µm filter (CellTrics^®^, Sysmex, Kobe, Japan) filter. To remove UV-fluorescing coumarin compounds – which have been reported for subsp. *vaseyana* and its hybrids (McArthur et al., 1988; Shultz, 2009) – 0.5 mL of 60% glycerol solution was added to each isolate, samples were centrifuged at 3,200 rpm for three minutes, and the supernatant was discarded; this treatment has been demonstrated to mitigate interference with the DAPI stain effectively (Kolář et al., 2012). The nuclei were then resuspended with 0.5 mL Nuclei Extraction Buffer (Sysmex, Kobe, Japan) and prepared for flow cytometry (CyStain UV Precise P Kit, Sysmex, Kobe, Japan). Individual genome size was estimated for at least five individuals per plot. Ploidy for the remaining individuals was estimated by pooling up to four individuals per plot, and comparing their relative fluorescence using a flow cytometer (CyFlow Space, Sysmex, Kobe, Japan). For each sample, measurements were taken at least twice to assess variation between measurements. Measurements were calculated as relative fluorescence (RF; sample mean/reference mean) due to inconsistent genome size reports for the reference taxon. Ploidy was then inferred based on the relationship between genome size and chromosome number as provided in Garcia et al. (2008).

### 2.4 ddRAD library preparation

A double-digest RAD library was prepared using EcoR1 and Mse1 restriction enzymes (New England Biolabs, Ipswitch, MA, USA), following the protocol of Parchman et al. (2012) with minor modifications. Specifically, to account for allele dosage, samples were pooled using twice the amount of PCR product for the tetraploids than for the diploids. To improve variant calling, 17 technical replicates were included. The final library was shipped to the Genomics & Cell Characterization Core Facility (GC3F) at the University of Oregon (https://gc3f.uoregon.edu/) for fragment size selection (Blue Pippin Prep, 400 bp to 550 bp; Sage Sciences Inc., Beverly, MA, USA) and sequencing using 150 bp paired-end reads (Illumina NovaSeq S4 lane, Illumina Inc., San Diego, CA, USA).

### 2.5 Variant calling

Adapter incompatibilities at the read-2 sequencing primer resulted in a lack of signal for the reverse reads; thus, only the forward reads were included in analyses. Raw sequenced reads were demultiplexed as single-end reads (process_radtags function of STACKS 2.60, Catchen et al., 2013) based on inline barcodes and filtering for uncalled bases (-c) and low quality (-q). The demultiplexed files were then filtered for polyG tails (-g), adapter content (-a), and a minimum length of 139 bp (fastp, Chen et al., 2018; available at https://github.com/OpenGene/fastp).

Each filtered read file was mapped against the *A. tridentata* reference genome (Melton et al., 2022), using the maximum exact match (MEM) algorithm (BWA 0.7.17, Li and Durbin, 2010). The resulting SAM files were sorted by reference coordinates and annotated with read groups (picard tools 2.9.2; available from https://broadinstitute.github.io/picard/). Due to the highly repetitive nature of the *A. tridentata* genome, only high-quality (mapQ ≥ 30) uniquely mapped reads were selected (SAMtools 1.15, Li et al., 2009) for downstream analysis. Variant calling and genotype estimation was performed (GATK HaplotypeCaller, Poplin et al., 2017) using the genomic variant call format mode (-ERC GVCF) for each individual and ploidy level (-ploidy 2 and -ploidy 4 for diploids and tetraploids, respectively). Files for each genomic region (i.e., chromosomes and unassigned scaffolds) were merged (GATK GenomicsDBimport), and joint genotyped (GATK GenotypeGVCF).

For subsequent analyses, we used the approach implemented in EBG 0.3.2 (Empirical Bayesian Genotyping in Polyploids; Blischak et al., 2018) which integrates position-specific sequencing error and a flat prior of genotypes to calculate ploidy-informed genotype likelihoods. We filtered data to retain only biallelic variants (filter-vcf.R script from EBG), allowing for a maximum of 10% missing individuals, a minimum quality of 100 (minQ>100), and a minimum depth of five (DP>5) per individual. We removed samples of alternate ploidy from each VCF file (custom R function), and selected for shared variants between the diploid and tetraploid datasets (intersect-vcf.R script from EBG). The quality-filtered BAM files and the mpileup function of SAMtools 1.15 (Li et al., 2009) were used to extract error rates for each of the retained variant positions with EBG’s per-locus-err.py script. Finally, ploidy-aware genotypes were inferred as derived allele counts with 10,000 iterations and the EBG GATK model. The EBG output was subsequently filtered for an alternate allele frequency of > 0.04 and converted into specific formats for further analyses using bash and R tidyverse packages (https://www.tidyverse.org/).

### 2.6 Population structure and hybridization in a heteroploid SNP dataset

To explore relationships between the heteroploid *A. tridentata* subspecies, and to infer the presence of hybridization and introgression, a relatedness coefficient matrix between individuals was calculated using a coancestry estimator based on that proposed by Ritland (1996) as implemented in PolyRelatedness 1.11b (Huang et al., 2014). The method-of-moments estimator of Ritland (1996) is particularly suitable for polyploid and mixed ploidy datasets, as shown by Ahmad et al. (2021) and Brandrud et al. (2020). The derived relatedness coefficients between non-relatives from the same gene pool are expected to approach zero and expected to approach one if an individual is compared to itself. Relatedness coefficients smaller than zero or larger than one are possible and represent statistical errors of inferring relatedness, caused by differential allele sharing or correction for ploidy (Ritland, 1996, Huang et al., 2014). The relatedness patterns were first visualized as a principal coordinates analysis (PCoA) by extracting eigenvectors (function eigen in R) and plotting the first vectors (ggplot2 3.3.6 in R). A comprehensive visualization of the relatedness matrix was generated as a heatmap (heatmap.2 function of gplots, available from https://cran.r-project.org/package=gplots). To infer putative diploid progenitors of the tetraploid lineages, relatedness values between tetraploids and their putative diploid parents were extracted, and tested for significance using a Wilcoxon test (ggsignif package in R, Ahlmann-Eltze and Patil, 2021).

Based on the EBG output, but including only one SNP per ddRAD locus to reduce linkage between markers and with modifications for a polyploid dataset outlined in Stift et al. (2019), we applied a model-based clustering approach (STRUCTURE 2.3.4, Pritchard et al., 2000) using a polyploid model, 100,000 replicates, and 30,000 burn-ins for values of K ranging from one to ten, repeating each run ten times. The results were imported into R in order to determine the best-fit K value (Evanno et al., 2005), as well as to plot the results (pophelper 2.2.9 package (available from: http://royfrancis.github.io/pophelper/). Samples within figures were colored in agreement with those assigned by STRUCTURE under the best-fit K value.

To assess inheritance patterns of the tetraploid populations – and thus, infer the mode of WGD – we utilized the filtered dataset (1,266 SNPs) and plotted genotype frequencies against allele frequencies for tetraploid samples not exhibiting introgression of q > 0.1 for each polyploid lineage (117 samples for *wyomingensis* 1 and 49 samples for *wyomingensis* 2), respectively. This approach – proposed by Lloyd and Bomblies (2016) – has been applied to RADseq datasets (Ahmad et al., 2021).

## 3 Results

### 3.1 Contemporary and future climate niche

**Figure 2:**
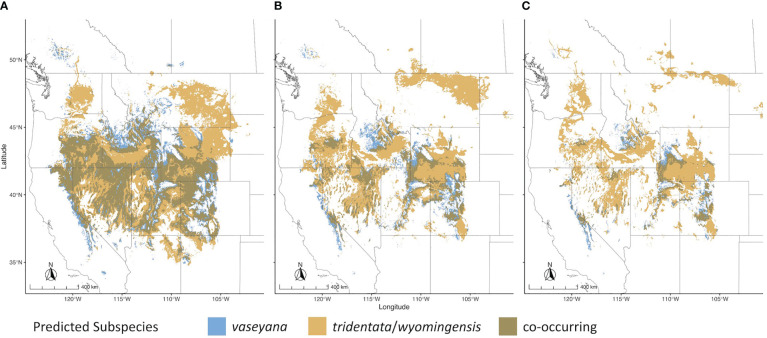
Range wide climatic niches for subspecies under different climate scenarios. **(A)** Contemporary climatic niche model; **(B)** 2050 RCP4.5 emission scenario; **(C)** 2050 RCP8.5 emission scenario.

For each of the climate niche models, seven climate variables were identified, six of which (AHM, bFFP, DD_0, MAP, MAR and MSP; **Table 1**) were consistent between the models, only differing by one variable (PAS for subsp. *vaseyana*, PPT_wt for subsp. *tridentata*/*wyomingensis*; **Table 1**). The contemporary model predicted habitat suitability for a total of 1.2 M km^2^ (**Table 2**) and predicted co-occurring subspecies in 44% of its contemporary range, including all five sampled transects (**Figure 1B**). The mid-century RCP4.5 and RCP8.5 models predicted suitable habitat for a total of 0.75 M km^2^ and 0.52 M km^2^, with co-occurring subspecies in 24% and 16%, respectively (**Figure 2** and **Table 2**).

**Table 1:** Climate variables used in niche modeling of *A. tridentata* subspecies as identified by the random Forest algorithm.

Acronym	Definition
AHM	annual heat-moisture index (MAT+10)/(MAP/1000)
bFFP	the day of the year on which frost free period begins
DD_0	degree-days below 0°C, chilling degree-days
MAP	mean annual precipitation (mm)
MAR	mean annual solar radiation (MJ m^-2^ d^-1^)
MSP	May to September precipitation (mm)
PAS	precipitation as snow (mm) between August in previous year and July in current year
PPT_wt	winter precipitation (mm)

**Table 2:** Suitable habitat for *A. tridentata* subspecies under contemporary and two mid-century climate scenarios as predicted by the climatic niche models as well as the proportion of lost habitat compared to the contemporary distribution.

Subspecies	Total Area (km^2^)			Percent Loss	
	contemporary	2050_RCP4.5	2050_RCP8.5	2050_RCP4.5	2050_RCP8.5
*vaseyana*	148,489	89,834	51,216	39.50	65.51
*wyoming/tridentata*	526,272	479,806	386,594	8.83	26.54
co-occurring	529,889	182,275	83,271	65.60	84.29
total	1,204,650	751,916	521,081	37.58	56.74

### 3.2 Ploidy assessment

Average RF-values (± SD) of 140 individually measured samples were 6.73 (± 0.15) for the diploid subsp. *tridentata*, 6.63 (± 0.24) for diploid subsp. *vaseyana* and 12.2 (± 0.49) for tetraploid subsp. *wyomingesis* (**Table 3**). A total of 203 samples were identified to be diploid, 213 were identified to be tetraploid. Four samples exhibited inconclusive RF values and were excluded from further analyses.

**Table 3:** Mean relative fluorescence (± SD) for each subspecies and the count of individually measured samples.

Subspecies	mean RF (± SD)	number of individuals
*tridentata*	6.73 ( ± 0.15)	18
*vaseyana*	6.63 ( ± 0.24)	50
*wyomingensis*	12.2 ( ± 0.49)	72

### 3.3 Sequencing and variant calling

After demultiplexing and filtering, a median (± SD) of 1.86 (± 0.61) million high-quality reads per diploid sample, and 3.68 (± 1.61) million high-quality reads per tetraploid sample were retained, 99.4% of which mapped to the reference genome. With the removal of reads mapped at multiple positions or with low confidence, a median (± SD) of 249,540 (± 107,626) and 555,929 (± 246,946) reads were retained per diploid and tetraploid sample, respectively.

After filtering for 10% missing data, minQ > 100 and DP > 5, the genotype estimation approach yielded information for 19,277 SNPs. Optimization and further exclusion of any variants with an alternate allele frequency smaller than 0.04 and selection of a single SNP per 139 bp window retained 1,266 and 540 SNPs, respectively.

### 3.4 Population structure and extent of hybridization

**Figure 3:**
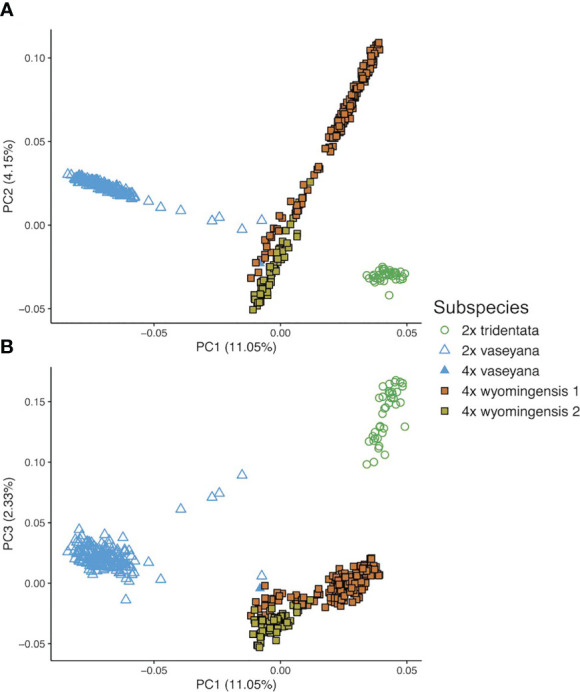
Genetic structure of *A*. *tridentata* extracted as Principal Coordinates (PC) based on pairwise relatedness. **(A)** shows PC1 and 2, **(B)** shows PC1 and 3.

The first three eigenvectors of the PCoA explained 17.5% of the variation. Principal Coordinate 1 showed a clear separation between the diploid subspecies, whereas polyploid individuals showed a clinal pattern in ancestry proportions between two groups, northern and southern (**Figure 3**). However, the results of the model-based clustering approach (see below) supported a division between these polyploid groups.

**Figure 4:**
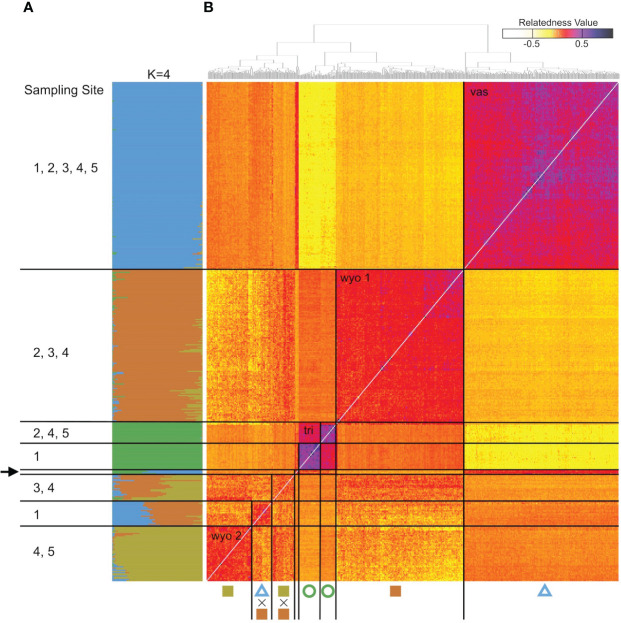
Genomic variation in *A*. *tridentata*. **(A)** Ancestry proportions bar plot as identified with STRUCTURE based on 540 SNPs and the four-population model (K=4). Each bar corresponds to an individual and each color to a genetic cluster. Individuals are sorted by the dendrogram inferred by the relatedness heatmap. **(B)** Pairwise relatedness heatmap based on 1,266 SNPs between 434 heteroploid individuals from 21 plots across the western United States. Numbers on the left indicate transect sites contained in a group, the arrow indicates the group containing diploid hybrids from various sites. The legend on the top right corresponds to the relatedness coefficients as estimated by Ritland’s method-of-moments estimator, the diagonal (i.e., individual compared to itself) was removed for improved contrast. Symbols below correspond to **Figure 1**.

**Figure 5:**
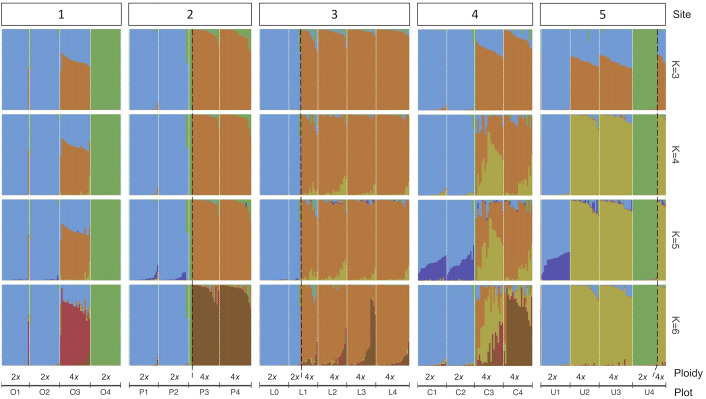
A genetic structure bar plot of 434 individuals inferred by 540 SNPs under different population models (K=3 to K=6). Plots within transect sites are arranged in decreasing elevation from left to right and separated with white dotted lines. Black dashed lines separate ploidy within a plot.

The analysis of the model-based clustering results indicated a model of four populations (K=4) best explained the genetic variation (**Supplementary Figure S1**). The bar plot (**Figures 4A**, **5**) and relatedness heatmap (**Figure 4B**) supported a clear separation between diploid subspecies as well as two distinct tetraploid groups. In the heatmap (**Figure 4B**), the diploid group of subsp. *vaseyana* appeared to be more homogeneously distributed across the sampling region (sites 1 to 5) compared to diploid subsp. *tridentata*, which exhibited a clear population structure corresponding to the sampling sites (1 and 2). Within the tetraploids, both the model-based clustering approach and the relatedness heatmap identified two groups, corresponding to the northeastern sampling locations (*wyomingensis* 1; sites 2 and 3) and the southernmost sampling location (*wyomingensis* 2; site 5). In addition, two sites (1 and 4) exhibited a high degree of admixture at both the homoploid (4) and the heteroploid (1) level. Specifically, a plot at the northwestern transect (1) shows a signal of mixed ancestry with subsp. *vaseyana*, which segregates into a separate cluster at K ≥ 6, which may represent a third origin of a tetraploid lineage in our dataset (**Supplementary Figure S2**).

Based on the clustering results and a 10% (q > 0.1) cut-off threshold, 2.5% of the diploid subsp. *tridentata* and subsp. *vaseyana* were identified to be introgressed. In contrast, 18.4% of the tetraploids showed signals of introgression from one of the diploid subspecies, and 17.6% of the tetraploids showed signals of gene flow between tetraploid groups (**Table 4**).

**Table 4:** Percentage of introgressed individuals per subspecies at three different q-thresholds.

Subspecies	introgressed by	q>0.05 (%)	q>0.1 (%)	q>0.15 (%)
*tridentata*	*vaseyana*	0.5	0.5	0.5
*vaseyana*	*tridentata*	2.4	2.0	2.0
*vaseyana*	*wyomingensis* 1	0.4	0.4	0.4
*wyomingensis* 1	*tridentata*	4.4	3.5	1.8
*wyomingensis* 1	*vaseyana*	15.9	12.3	10.6
*wyomingensis* 1	*wyomingensis* 2	15.0	12.3	7.9
*wyomingensis* 2	*tridentata*	1.3	0.4	0.4
*wyomingensis* 2	*vaseyana*	4.0	2.2	0.4
*wyomingensis* 2	*wyomingensis* 1	5.3	5.3	5.3

### 3.5 Inheritance mode and polyploid formation

**Figure 6:**
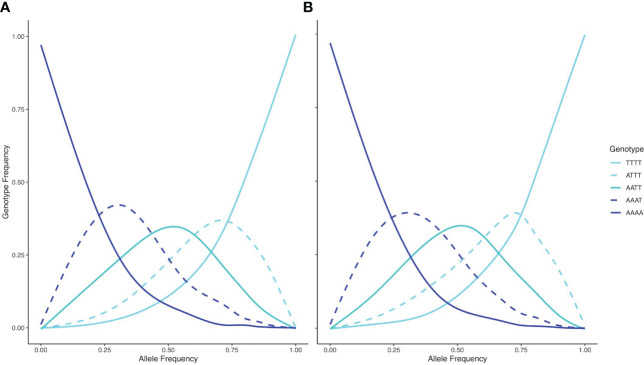
Relationships between genotype frequencies (y-axis) and allele frequencies (x-axis) for based on 1266 SNPs and 117 samples for wyomingensis 1 **(A)** and 49 samples for *wyomingensis* 2 **(B)**. Both lineages exhibit all five possible genotypes at an allele frequency of 0.5, which is consistent with a tetrasomic inheritance pattern.

**Figure 7:**
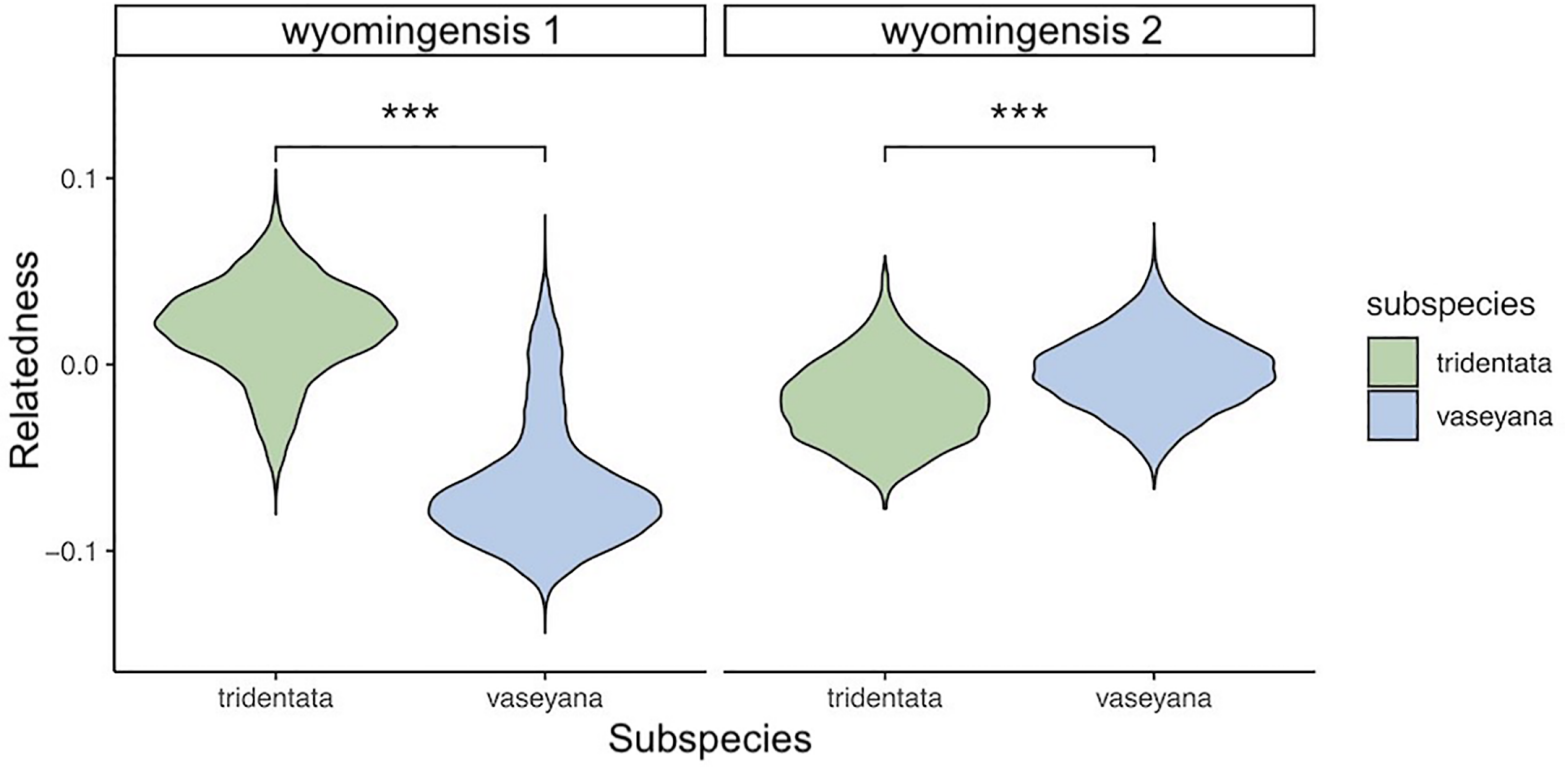
Relationship between two tetraploid lineages (*wyomingensis* 1 and *wyomingensis* 2) and putative diploid progenitors as extracted from the pairwise relatedness heatmap. Asterisks (***) indicate significant at p < 0.001.

The inheritance pattern of natural populations – and therefore the mode of WGD (auto- vs. allopolyploidy) can be distinguished by the genotype frequencies of biallelic loci. Populations exhibiting all five possible genotypes (i.e., AAAA, AAAT, AATT, ATTT, TTTT) at an allele frequency of 0.5 indicates random segregation patterns between subgenomes, as observed in autopolyploids. Conversely, a population exhibiting a duplex heterozygous genotype (i.e., AATT) in all individuals at an allele frequency of 0.5 indicates disomic inheritance, as observed in allopolyploids (Lloyd and Bomblies, 2016). The relationship between allele and genotype frequencies for tetraploid populations in this study showed five observable genotypes at an allele frequency of 0.5, indicating a tetrasomic inheritance, which is consistent with a scenario of autopolyploidy (**Figure 6**). Testing both tetraploid gene pools for differential relatedness with their diploid progenitors reveals significantly (p < 0.001) higher relatedness of *wyomingensis* 1 with subsp. *tridentata* and *wyomingensis* 2 with subsp. *vaseyana* (**Figure 7**).

## 4 Discussion

### 4.1 Subspecies separation and hybridization

The heteroploid *A. tridentata* subspecies in our sampling areas were consistently separated into four genetic groups that differ by subspecies classification, ecology, ploidy and geography. In accordance with our expectations and findings from a previous study (Richardson et al., 2012), the diploid populations (i.e., subsp. *tridentata* and subsp. *vaseyana*), form two distinct groups, corresponding to the currently recognized subspecies. Subspecies *tridentata* exhibits population structure corresponding to the two main sampling locations (1 and 5), which is in agreement with a scenario of isolation by distance (IBD; **Figure 4B**; **Supplementary Figure S3**), whereas subsp. *vaseyana* appears genetically well-mixed and did not exhibit population structure across all five transects. Population structure was not inferred for this subspecies complex by Richardson et al. (2012) but may be explained by unrestricted pollen flow in the open, higher-elevation habitats that subsp. *vaseyana* occupies compared to the topographically more structured basins that subsp. *tridentata* occupies.

In accordance with our hypotheses, the tetraploids formed two major groups: one which corresponded to the northeastern transects (2 and 3; **Figure 4A**), and a second that corresponded to the southern transect (5; **Figure 4A**). The tetraploid population, sampled from the transect at the Idaho-Utah border (4; **Figure 4A**), contained individuals from both tetraploid populations – as well as hybrids between them – indicating secondary contact between the two distinct tetraploid lineages at this transect. A tetraploid population from eastern Oregon (1; **Figure 4A**) appeared to be admixed between the northern tetraploid lineage and the diploid subsp. *vaseyana* (q > 0.3) gene pool: at higher levels of K (≥ 6), this population was assigned to its own gene pool while still exhibiting introgression from subsp. *vaseyana*, although at lower proportions (**Figure 5**; **Supplementary Figure S2**).

In contrast to our expectations, signals of hybridization between diploid subspecies (i.e., q ≥ 10% of either gene pool) were detected in 2.5% of all diploid individuals. All the introgressed diploid individuals shared relatively high admixture proportions (q > 0.3) throughout all runs of the model-based clustering approach, indicating recent formation. Hybridization was determined to predominantly occur unidirectionally from subsp. *vaseyana* to subsp. *tridentata* (**Figure 4A**; **Table 4**). Strong reproductive barriers between genomically distinct taxa (Comeault and Matute, 2018; Lavania, 2020) or the tension zone model of hybridization (Barton and Hewitt, 1985), which assumes low hybrid fitness, may explain the low number of diploid hybrids. Conversely, the ecotone model assumes hybrid superiority along environmental gradients and was previously suggested for *A. tridentata* (McArthur et al., 1988; Abbott and Brennan, 2014), but was not identified here. However, areas of hybridization between *A. tridentata* subspecies may be limited to narrowly defined ecological niches, which we did not capture in our transects. Previously sampled transects – which reported a high number of putative hybrids based on morphological and chemical markers (McArthur et al., 1988) – were less than 0.1 km compared to our transects ranging from 6 km to 15 km (**Figure 1B**).

Tetraploid populations overall exhibited a higher fraction of individuals (~18% at q > 0.1) introgressed from either of the diploid lineages, which is possible *via* unreduced gametes (Kreiner et al., 2017) and may contribute to an adaptive advantage for polyploid individuals (Schmickl and Yant, 2021). The northern tetraploid lineage *wyomingensis* 1 (sites 2 and 3) showed introgression from both diploid progenitors, while the southern tetraploid lineage *wyomingensis* 2 (site 5) showed almost introgression predominantly from diploid subsp. *vaseyana*, respectively (**Figure 4A**). Gene flow between tetraploid groups was detected in ~17% of the tetraploid individuals and exhibited the highest admixture proportions at site 4, which represents the central transect between the northern and southern transects. This pattern was consistently inferred throughout all model-based clustering runs (**Figures 5**, **S2** and **Table 4**).

Low levels of hybridization between diploids, but increased hybridization in tetraploids were also found in diploid-autotetraploid members of the genus *Arabidopsis* (Marburger et al., 2019; Monnahan et al., 2019). This pattern was termed “WGD-mediated gene flow” (Schmickl and Yant, 2021) and is applicable to *A. tridentata*, as we found a seven-fold larger proportion of admixed tetraploid individuals, compared to the admixed diploid individuals (**Table 4**). This indicates a breakdown of reproductive barriers at the tetraploid level and may increase introgression and potentially enhance adaptation to diverse and new environments (Schmickl and Yant, 2021; Buck et al., 2022).

### 4.2 Independent polyploid origins and different sources for WGD

Several different ploidy levels are well documented in the genus *Artemisia* in general and in the subgenus *Tridentatae* specifically (McArthur and Sanderson, 1999; Pellicer et al., 2010). In *A. tridentata*, polyploid populations have been widely found and are abundant across its distribution (McArthur and Sanderson, 1999). Recent studies suggest independent origins of the tetraploids (Richardson et al., 2012), as well as cycles of polyploidization and possible re-diploidization (Melton et al., 2022). These findings indicate that WGD is an evolutionary mechanism and adaptive process of ecological importance in this taxonomic group. The frequency and abundance of polyploid populations across the landscape raises the question of whether there were one or more WGD events, and whether they arose within a diploid lineage (autopolyploidy), or as a consequence of hybridization, followed by WGD (allopolyploidy). In contrast to our expectations, the model-based clustering analysis consistently inferred two (K=3 to K=5) or four (K≥6) distinct tetraploid gene pools (**Figure 5**), indicating at least two independent origins. We identified *wyomingensis* 1, associated with the central and eastern transects (2 and 3) and *wyomingensis* 2, associated with the southernmost transect (5). These two lineages may have formed independently during the Pleistocene, where pluvial lakes and unsuitable climatic conditions in the Snake River Plain may have acted as barriers and restricted gene flow between populations (Davis et al., 1986; Houston et al., 2014).

Inferring the mode of inheritance showed that all genotypes were present at an allele frequency of 0.5 and that an excess of the duplex heterozygous genotype could not be observed. This suggests tetrasomic inheritance, which is consistent with an autopolyploid origin for the tetraploid lineage, or an allopolyploid origin with widespread recombination between parental subgenomes (**Figure 6**). Within population WGD agrees with previous studies in *A*. *tridentata* (McArthur et al., 1981; McArthur et al., 1988), and could have been triggered by temperature oscillations during the Pleistocene, which may have caused an increased frequency of unreduced gametes and independent autopolyploid formation (Kreiner et al., 2017). However, some studies (Meirmans and Van Tienderen, 2013; Blischak et al., 2022) suggest that chromosomal segregation patterns follow a continuum rather than strict di- or tetrasomic inheritance. Additionally, in neo-allopolyploids, higher rates of homeologous recombination are possible, leading to homogenization of sub-genomes (Lloyd and Bomblies, 2016) and even a low frequency of allelic exchange between subgenomes may homogenize allele frequencies (Meirmans and Van Tienderen, 2013) and mask an allopolyploid origin even though those lineages are expected to exhibit still noticeably different allele frequencies than those of autopolyploids (Lloyd and Bomblies, 2016). Thus, WGD in the tetraploid lineages identified here may still have been induced by hybridization, but patterns of allele frequencies may have been masked by recombination. Demographic inference of the tetraploid lineages may improve our understanding of polyploid formation in *A. tridentata* but was deemed to be unsuitable with our dataset (see below). Relationships between tetraploid lineages and their diploid progenitors appeared to be different for each tetraploid lineage, where subsp. *tridentata* exhibited closer relatedness with *wyomingensis* 1, while subsp. *vaseyana* was more closely related to *wyomingensis* 2 (**Figures 4B**, **7**), further supporting independent origins. One tetraploid population at site 1 appeared to contain large proportions of both, *wyomingensis* 1 and subsp. *vaseyana*. The comparison of the relatedness values confirms this close relationship. Thus, indicating either recent hybridization, or contribution to the tetraploid formation. At K ≥ 6, this population segregated into a separate gene pool (**Figure 4**, **Supplementary Figure S2**), potentially indicating a third distinct origin in our dataset. However, if mixing among tetraploid lineages is relatively common, it would act to obscure multiple origins of tetraploid populations in a genomic analysis.

### 4.3 Climate change, subspecies distribution and potential for hybridization

The *A. tridentata* range, once covering over 6 million km^2^ in the western United States, has faced a drastic decline due to human land use, increased wildfire frequency and climate change, causing an estimated loss of ~44% of its historic range (Davies et al., 2011). Based on our climate niche model under the mid-century RCP4.5 and RCP8.5 scenarios, climate alone may cause an additional loss ranging from 38% to 57% of the total contemporary distribution (**Table 2**, **Figure 2**). The climatically suitable regions for all subspecies to co-occur may decrease by >65% by 2050 (**Table 2**), a stark reduction which would limit the potential for hybridization between subspecies and consequently limit the generation of novel genotypes, phenotypes, and potentially WGD.

Habitat suitability for the climatically more specialized subsp. *vaseyana* was modeled to be reduced by 39% to 65% (**Table 2**) of its contemporary range, losing large areas in the Great Basin region and restricting its range to higher altitudes, such as the Sierra Nevada and the Rocky Mountains (**Figure 2**). The subspecies least affected by climate change in our model were subsp. *tridentata*/*wyomingensis*; their ranges were predicted to be reduced by 25% in the highest emission scenario and 8% in the moderate scenario (**Table 2**). The RCP8.5 estimate is similar to range reductions reported in subsp. *wyomingensis* by Still and Richardson (2015) at 39%. While both subsp. *tridentata* and subsp. *wyomingensis* are adapted to similar climates, subsp. *wyomingensis* is better adapted to the dry and hot climates of the western United States and is more robust during drought due to its different soil requirements (Kolb and Sperry, 1999). Thus, subsp. *wyomingensis* has an advantage over other subspecies under increasing temperature scenarios, particularly if scenarios of RCP4.5 or lower can be met. Additionally, the tetraploid subsp. *wyomingensis* may benefit from neo- or subfunctionalization of genes which may allow for a faster adaptation to changing climates (Van de Peer et al., 2017; Van de Peer et al., 2021) and WGD-mediated gene flow may additionally increase the adaptive potential of the tetraploids, mitigating negative effects of climate change (Schmickl and Yant, 2021).

The success of *A. tridentata* across its range was – partly – attributed to a reticulate evolutionary pathway and stable hybrid zones were expected to serve as a reservoir for fit hybrids (McArthur et al., 1988; McArthur and Sanderson, 1999). We did not find evidence for extensive hybridization, but argue that maintaining sympatric populations is important to conserve genetic diversity, as hybridization may contribute to the adaptive divergence of populations (Allendorf et al., 2001; Janes and Hamilton, 2017). The latter was demonstrated in North American tree species, where hybridization in sympatric regions gave rise to drought-adapted lineages, while allopatric populations remained genetically distinct (Buck et al., 2022), highlighting the importance of conserving hybrids in the light of climate change.

### 4.4 ddRAD sequencing and variant calling

We performed a ddRAD-sequencing approach (Parchman et al., 2012) to identify population structure and the extent of hybridization in a non-model organism with a large and complex genome, which ddRAD-sequencing has successfully been applied to (Parchman et al., 2012; Peterson et al., 2012; Andrews et al., 2016; Faske et al., 2021). Recently developed methods implementing genotype likelihoods have been shown to counteract genotyping uncertainties in particular for high throughput sequencing datasets of low to intermediate coverage, including those generated by (dd)RADseq (Blischak et al., 2018; Warmuth and Ellegren, 2019). In the case of complex polyploid genomes, their variety of recombination and segregation patterns requires higher coverage, which has additional benefits when downstream data analyses are based on genotype likelihoods. However, we want to highlight caveats that may arise by combining original wet-lab protocols with current high-throughput sequencing strategies.

Incompatibilities between index and sequencing primers in the adapter sequences may cause the loss of reverse reads (**Supplementary Figure S4**). Additionally, for large and repetitive genomes, the use of AT-rich restriction enzymes, such as EcoR1 and Mse1, may enrich repetitive elements and reduce the number of informative loci (Macas et al., 2002). Furthermore, the application of a high number of PCR cycles may not only increase the number of single-stranded DNA through reagent depletion, which negatively impacts size selection, but may also increase the number of PCR errors. The latter subsequently biases downstream analyses (Robasky et al., 2014; Potapov and Ong, 2017), particularly influencing minor allele frequency (MAF; Ma et al., 2019), and complicates the assessment of population structure and demographic inference (Linck and Battey, 2019; Warmuth and Ellegren, 2019). The use of methylation-sensitive restriction enzymes may prevent restriction of heterochromatic regions and result in a higher number of uniquely mapped reads (Parchman et al., 2012; Pootakham et al., 2016). Increasing the amount of starting DNA and reducing PCR cycles may decrease PCR errors (Potapov and Ong, 2017). Furthermore, the use of unique molecular identifiers (Kivioja et al., 2012) allows removal of PCR duplicates, which could bias read coverage and cause allele dropout or false positive variant calls (Schweyen et al., 2014; Zverinova and Guryev, 2022).

## 5 Conclusions

Our objectives were to investigate the genetic distinctiveness and extent of hybridization among three *A. tridentata* subspecies at different ploidies, to identify the origins of polyploidy in these taxa, and to predict the extent of range overlap and potential for hybridization under contemporary and future climate scenarios. We found that diploid subspecies are genetically distinct lineages and diploid hybrids between these lineages are rare. Similarly, we discovered at least two distinct tetraploid lineages, which in contrast to the diploids, exhibit signals of hybridization both at the homoploid and heteroploid level. Furthermore, tetraploids appear to be of independent autopolyploid origins, each of which may have been formed by a different parental diploid congener. Lastly, our climate niche models predict a loss of suitable climatic niches, causing regions of subspecies range overlap to decrease, reducing the potential for hybridization. Our results underscore the value of conserving and restoring big sagebrush ecotones to maintain generation of genetic diversity and polyploidy, an important source of adaptive genetic variation.

## Data availability statement

The unfiltered, demultiplexed reads can be found at NCBI (https://www.ncbi.nlm.nih.gov) under the accession number PRJNA942958. The genotype matrix and the code for data conversion and analysis prepared for this study can be found at https://github.com/LukeBotanist/Sagebrush.git.

## Author contributions

LG, PH, LW, and BR designed and planned the study. EM generated the climate niche models. EM and LG performed geospatial analysis. BR and LG selected sites and collected samples. LG performed flow cytometry, prepared the ddRAD-seq library and analyzed the data. LG, PH, LW, and BR discussed and interpreted the results. LG and BR wrote the manuscript with input from all authors. All authors contributed to the article and approved the final version.
