# Supplementary material for: Population structure and hybridization under contemporary and future climates in a heteroploid foundational shrub species (Artemisia tridentata)
Source: Front Plant Sci. 2023 May 22;14:1155868. doi: 10.3389/fpls.2023.1155868 (PMC10239881; doi:10.3389/fpls.2023.1155868)
Supplement: Supplementary file 2 [file DataSheet_1.docx]

Supplementary Material

# Supplementary Methods

To test for isolation by distance (IBD), Ritland’s method of moments based pairwise relatedness values were subtracted from one (1-relatedness coefficient) to convert the relatedness matrix to a distance matrix. A pairwise geographic distance matrix was calculated using the distm function as implemented in the package geosphere 1.5-18 (Hijmans, 2022) in R. The matrices were then tested for correlation and significance using the mantel function as implemented in vegan 2.6-4 in R, using a Pearson correlation.

# Supplementary Figures

# Supplementary Figure S. Results of STRUCTURE analysis as inferred with pophelper 2.2.9 based on Evanno’s method (Evanno, 2005). (A) Mean log-normal probability (±SD) and (B) Evanno’s Δ*K* in relation to the inferred clusters based on ten replicate runs per cluster.

**Supplementary Figure S2**. Genetic structure of 434 individuals inferred by 540 SNPs under different population models (K=2 to K=10). Plots within transect sites are arranged in decreasing elevation from left to right and separated with white dotted lines.

Supplementary Figure Relationship between genetic distance (1 - relatedness coefficient; y-axis) and geographic distance (x-axis). The inferred pattern is consistent with a scenario of isolation by distance (IBD).

Supplementary Figure S Schematic of the ddRAD sequencing approach following Parchman et al. (2012). Adaptors include adaptor sequence, barcode, cutsite and protector base. Adaptors in color; EcoR1 on the left, Mse1 on the right. The highlighted sequence show the index 1 primer and the read 2 sequencing primer, which corresponds to the reverse complement of the index 1 primer which may cause failure in sequencing the reverse reads. Figure adapted from Parchman et al. (2012), supplementary information RF_seq_protocol available at <https://datadryad.org/stash/dataset/doi:10.5061/dryad.m2271pf1>

# Supplementary Table

Table S1 is submitted as excel file.

**Table S1**. List of all samples included in this study and the corresponding metadata. The latter includes identifiers for each sample, the Stillinger herbarium (ID) and the NCBI Biosamples; assigned subspecies, experiments performed, flow cytometry (FCM) information and sampling location. Subspecies were assigned based on the results of the model-based clustering approach (STRUCTURE) under the best-fit model (K=4). Ploidy was assigned based on the relative fluorescence values (RF) and relationships between genome size and chromosome number inferred from Garcia et al. (2008).

# Supplementary References

Garcia, S., Canela, M. Á., Garnatje, T., Mcarthur, E. D., Pellicer, J., Sanderson, S. C., et al. (2008). Evolutionary and ecological implications of genome size in the North American endemic sagebrushes and allies (Artemisia, Asteraceae). Biol. J. Linn. Soc. 94, 631–649. doi: 10.1111/j.1095-8312.2008.01001.x.

Hijmans R (2022). geosphere: Spherical Trigonometry. R package version 1.5-18, <https://CRAN.R-project.org/package=geosphere>

Oksanen J, Simpson G, Blanchet F, Kindt R, Legendre P, Minchin P, O'Hara, R, Solymos P, Stevens M, Szoecs E, Wagner H, Barbour M, Bedward M, Bolker, B, Borcard D, Carvalho G, Chirico M, De Caceres M, Durand S, Evangelista, H, FitzJohn R, Friendly M, Furneaux B, Hannigan G, Hill M, Lahti L, McGlinn D, Ouellette M, Ribeiro Cunha E, Smith T, Stier A, Ter Braak C, Weedon J (2022). vegan: Community Ecology Package. R package version 2.6-4, <https://CRAN.R-project.org/package=vegan>.

Parchman, T. L., Gompert, Z., Mudge, J., Schilkey, F. D., Benkman, C. W., and Buerkle, C. A. (2012). Genome-wide association genetics of an adaptive trait in lodgepole pine: ASSOCIATION MAPPING OF SEROTINY. *Molecular Ecology* 21, 2991–3005. doi: [10.1111/j.1365-294X.2012.05513.x](https://doi.org/10.1111/j.1365-294X.2012.05513.x).
